# Biological Properties and Medical Applications of Carbonate Apatite: A Systematic Review

**DOI:** 10.3390/pharmaceutics16020291

**Published:** 2024-02-18

**Authors:** Ralitsa Yotsova, Stefan Peev

**Affiliations:** 1 Department of Oral Surgery, Faculty of Dental Medicine, Medical University of Varna, bul. Tsar Osvoboditel 84, 9002 Varna, Bulgaria; 2 Department of Periodontology and Dental Implantology, Faculty of Dental Medicine, Medical University of Varna, bul. Tsar Osvoboditel 84, 9002 Varna, Bulgaria; stefan.peev@mu-varna.bg

**Keywords:** carbonate apatite, bone substitute, biomaterial, scaffold, tissue engineering, drug carrier

## Abstract

Bone defects represent an everyday challenge for clinicians who work in the fields of orthopedic surgery, maxillofacial and oral surgery, otorhinolaryngology, and dental implantology. Various bone substitutes have been developed and utilized, according to the needs of bone reconstructive surgery. Carbonate apatite has gained popularity in recent years, due to its excellent tissue behavior and osteoconductive potential. This systematic review aims to evaluate the role of carbonate apatite in bone reconstructive surgery and tissue engineering, analyze its advantages and limitations, and suggest further directions for research and development. The Web of Science, PubMed, and Scopus electronic databases were searched for relevant review articles, published from January 2014 to 21 July 2023. The study was conducted in accordance with the Preferred Reporting Items for Systematic Reviews and Meta-Analyses (PRISMA) guidelines. Eighteen studies were included in the present review. The biological properties and medical applications of carbonate apatite (CO_3_Ap) are discussed and evaluated. The majority of articles demonstrated that CO_3_Ap has excellent biocompatibility, resorbability, and osteoconductivity. Furthermore, it resembles bone tissue and causes minimal immunological reactions. Therefore, it may be successfully utilized in various medical applications, such as bone substitution, scaffolding, implant coating, drug delivery, and tissue engineering.

## 1. Introduction

Bone reconstruction surgery has rapidly developed in recent years, due to the advancements in tissue engineering, nanotechnology, and biomaterials science. The aim of bone regeneration is no longer a passive reconstruction with biocompatible and osteoconductive materials but, instead, the utilization of smart and stimuli-responsive biomaterials that closely resemble natural bone, which can guide stem cells for tissue regeneration, and act as drug delivery systems according to the patient’s individual needs [1,2,3].

The regeneration and repair of osseous defects caused by bone disorders, trauma, infections, and tumors represent an everyday challenge for clinicians in the fields of orthopedic surgery, maxillofacial and oral surgery, otorhinolaryngology, plastic surgery, cardiothoracic surgery, and dental implantology [4].

Various bone grafting materials have been developed and utilized according to the needs of bone reconstructive surgery [5,6], and the principles of guided bone regeneration usually require their application together with barrier membranes [7].

Numerous classifications of bone grafts have been suggested over the years [8,9,10]. According to their origin, they are generally divided into autografts, allografts, xenografts, and alloplastic materials [11]. In 2022, a novel enriched classification was introduced [12].

Autogenous bone grafts with their excellent biological properties are still considered the gold standard for the treatment of bone defects [13]. However, their utilization requires a second surgical site (donor site) and is related to increased post-operative morbidity. In addition, only a limited amount of bone can be harvested [14]. Alternatives to autogenous bone are allografts and xenografts; however, they were shown to possess inferior biological properties to autografts and may cause immunologic responses or infectious disease [14,15]. Xenografts and alloplastic materials have been preferentially used in recent years, due to their accessibility [16]. Several biomaterials are currently available for these purposes, such as demineralized freeze-dried bovine bone, deproteinized bovine bone mineral, natural coral, bioactive glass, and calcium phosphate ceramics (CPCs), including hydroxyapatite (HA), α-tricalcium phosphate (α-TCP), β-tricalcium phosphate (β-TCP), and biphasic tricalcium phosphate. Each biomaterial has its own advantages and limitations [17].

Calcium phosphate ceramics are widely utilized in bone reconstructive surgery as single materials or as biphasic calcium phosphate ceramics (BCPCs) [18,19]. Although HA has demonstrated great biocompatibility and osteoconductive potential, it acts as a foreign body in the bone and, thus, could increase the risk of infection [20,21]. In comparison, the resorption rate of β-TCP is much faster and exceeds the rate of bone deposition, which has led to its utilization in combination with HA [22].

The objective of biomaterials research and fabrication is to replicate natural tissues and their properties. The initial goal of tissue engineering is a simulation of the mechanical and chemical properties of the tissues, in order to restore their functionality, while the ultimate goal is the fabrication of materials that promote tissue regeneration and can serve as structural scaffolds, carriers, and drug delivery systems [23].

Successful biomaterials should possess the following biological properties: biocompatibility, bioinertness, bioactivity, and bioresorbability. They should also resemble natural bone and promote osteoinduction and osteoconduction [24].

Metal implants are usually used to fulfill the demands for strength; however, they do not have osteoinductive and osteoconductive properties. A transitional phase is needed to create a stable bond between the implant and the surrounding bone. Therefore, apatitic coatings are utilized to increase the bond at the bone–implant interface [25,26]. Apatites that are used as biomaterials should have appropriate physicochemical properties (e.g., surface properties, composition, porosity, particle size, solubility in a physiological environment), biological properties, and mechanical properties (e.g., hardness, stiffness, wear resistance) [24,27].

Mineralization strategies are regarded as a successful method for the development of biomimetic materials. Mineralized scaffolds resemble the inorganic component of natural bone. They enhance bone regeneration and demonstrate improved properties, which makes them a material with high potential in tissue engineering [28].

The term “biological apatite” is used to describe the HA in bone and teeth (e.g., enamel, dentin, cementum). The International Mineralogical Association’s Commission has adapted the Minerals Nomenclature and Classification, according to which biological apatite is a type of HA (more precisely, carbonated HA) [29].

According to Kono et al., more than half of the hydroxide and phosphate ions in apatite should be substituted with carbonate ions such that it can be referred to as “carbonate apatite”. Nevertheless, such a substitution has not been reported. Therefore, the authors suggest that biological apatite should be called “carbonated hydroxyapatite” instead of “carbonate apatite” [29].

Carbonate could substitute phosphate (B-type) and hydroxide (A-type) and, thus, alter the crystal properties of the material. Carbonate substitution in bone is usually B-type. While concomitant A- and B-type substitution has been reported, the A-type is rare in biological apatites compared to the synthetic representatives [30].

Madupalli et al. [30] prepared AB-type carbonated apatites with variable carbonate content and evaluated them using Fourier transform infrared spectroscopy (FTIR), powder X-ray diffraction, and carbonate assessment. The authors found that the two sites for substitution influence the crystal and domain sizes, as well as the material properties.

Several authors have demonstrated that key prerequisites for osteoconductivity and bone formation rate are the carbonate content of the graft, their porous structure, and the interconnectivity of the pores [31,32,33,34]. The porosity of the material provides an increased surface area and vascularization [33].

To increase the chemical resemblance to natural bone, carbonate was added to calcium-deficient hydroxyapatite (CDHA) through a novel biomimetic approach [35]. Carbonate ions increased the chemical reactivity of the apatites and fostered osteoclastogenesis [36,37,38].

In 2019, Barba et al. evaluated the impacts that the carbonate content and nanocrystal structure of biomimetic apatite have on bone regeneration. They used CDHA scaffolds in canine models and found that carbonate doping of the material promoted osteoinduction and bone regeneration. These findings suggest that the fabrication of bone substitutes with appropriate nanostructural and chemical features could allow for their use in natural bone remodeling [39].

Faster healing and increased bone regeneration of carbonate apatite (CO_3_Ap) were observed when compared to HA, β-TCP, and deproteinized bovine bone [40], indicating that materials with a composition that resembles natural bone could also demonstrate properties similar to bone [40,41].

The present review focuses on the use of CO_3_Ap in bone reconstructive surgery, with the aim of summarizing the current knowledge regarding its medical applications; discussing its biological properties, advantages, and limitations; and providing some recommendations for future research and developments.

CO_3_Ap has recently become a subject of increased interest for researchers in the fields of bone regeneration and materials sciences. It has reached the stage of clinical trials and has, so far, demonstrated promising biological properties. However, some further research is required to confirm its role in bone regeneration and tissue engineering, as well as its supposed superiority over the currently used bioceramics.

To the best of our knowledge, this is the first systematic review of articles discussing both the biological properties and medical applications of CO_3_Ap. This study aims to draw the attention of researchers in the field to this material which has, so far, demonstrated promising properties. Further investigations and clinical trials could prove or reject its superiority over the materials that are commonly or recently used for bone regeneration.

## 2. Materials and Methods

Ethical approval for this article is not applicable as it is inclusively based on the previously published literature.

### 2.1. Eligibility Criteria

The search included only review articles in English, published in the past 10 years (2014–2023), and containing the selected keywords. The inclusion criteria were articles that evaluated the biological properties and/or medical applications of CO_3_Ap in bone reconstruction and regeneration. The exclusion criteria were as follows: articles that are not reviews, case reports, and abstracts; studies that did not discuss CO_3_Ap’s biological properties and observed only its non-medical applications; articles before 2013; and articles in languages different from English.

### 2.2. Information Sources

This systematic review was conducted in accordance with the Preferred Reporting Items for Systematic Reviews and Meta-Analyses (PRISMA) Statement [42,43].

A comprehensive search for review articles in several electronic databases (Google Scholar, Web of Science, PubMed, and Scopus) was carried out on 21 July 2023.

### 2.3. Search Strategy

Only full-sized review articles written in English were included. The electronic search strategy comprised an advanced search in the selected databases:

1. In the Web of Science database, the following keywords were used: (carbonate apatite OR carbonated apatite) AND (bone substitute OR bone regeneration OR bone replacement);
2. In the PubMed database: carbonate AND apatite AND (bone AND substitute OR bone AND regeneration OR bone AND replacement);
3. In the Scopus database: carbonate AND apatite AND (bone AND (substitute OR regeneration OR replacement));
4. In Google Scholar, a precise search was not possible. The selected keywords were “carbonate apatite bone” with at least one of the words “regeneration”, “replacement”, or “substitution”.

### 2.4. Study Selection and Data Collection Process

Titles and abstracts were screened and evaluated for eligibility by two independent reviewers. As the advanced search in Google Scholar does not provide a precise sorting of articles with all the inclusion criteria, as was the case for the advanced search in the other three databases, only the results from Web of Science, PubMed, and Scopus were finally evaluated. The titles, abstracts, authors’ names, journal names, and years of publication of the studies were exported to an MS Excel spreadsheet, and duplicate records were removed. Then, the full-text articles were subjected to the above-mentioned inclusion and exclusion criteria. Discrepancies between the reviewers were resolved by discussion until a consensus was reached.

## 3. Results

**Figure 1:**
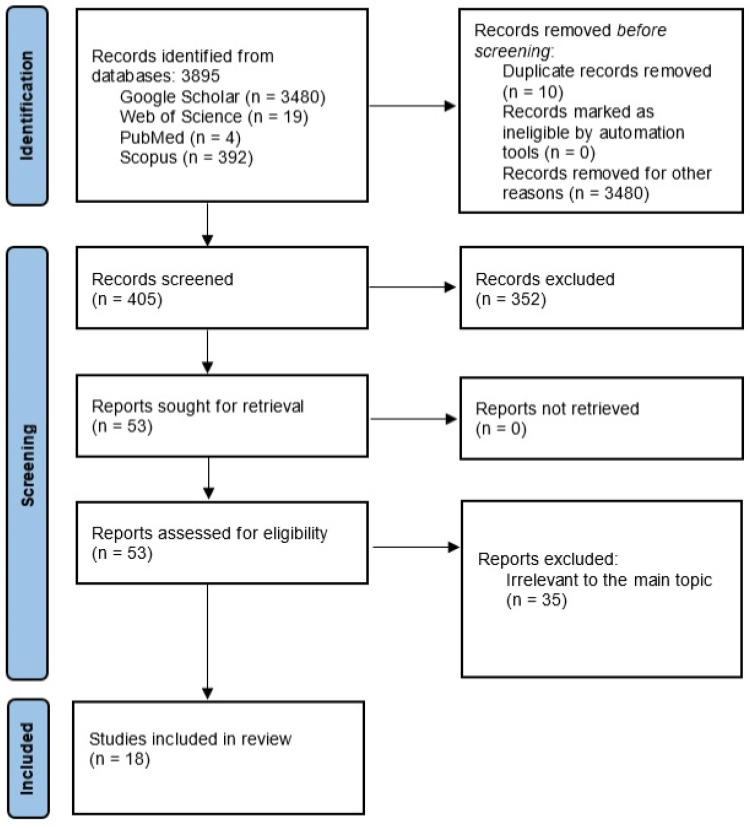
PRISMA flow diagram of the research.

The initial search identified 3895 potentially relevant review articles from the four databases over the last ten years. After the exclusion of the records from Google Scholar, due to the inability to advance the search with all the inclusion criteria and the irrelevance of the suggested articles, 415 studies remained. Ten duplicate records were excluded. This left 405 studies for evaluation. Finally, 18 studies relevant to the topic were included in the present systematic review. Figure 1 illustrates a PRISMA flow chart of the selection process.

Table 1 presents the characteristics of the studies included in this systematic review.

All the biological properties and medical applications listed in Table 1 were supported by in vitro trials, in vivo trials, one ex ovo trial, and human clinical trials. The in vivo studies were based on animal models, including rat, rabbit, dog, and sheep models. Although several review articles included in this study were based on a small amount of evidence, the overall conclusion about the biological properties and medical applications of CO_3_Ap coincided with the rest of the literature data, based on more in vitro and in vivo experiments.

### 3.1. Biological Apatites

From a chemical aspect, bone tissue is a composite material whose mineral component is calcium-deficient and non-stoichiometric apatite [21]. A widespread misconception in the medical field is that hydroxyapatite is the mineral phase of bones and teeth. For decades, experiments have been conducted to develop CO_3_Ap that resembles bone apatite and has similar properties [24,89,90].

Bone tissue regularly undergoes a stress-induced remodeling process. First, osteoclasts dissolve small amounts of bone tissue (collagen and apatite), which is then replaced through the deposition of new bone by osteoblasts [24,91]. Therefore, the apatite should be reactive under the acidic biological conditions created by the osteoclasts [24].

Although there is a chemical similarity between synthetic HA and natural bone, its capability to replace bone apatite is limited [92]. This significant drawback could be related to the absence of osteoinductive and antibacterial properties, low degradability, poor mechanical properties, and so on [93]. On the other hand, biological properties are related to physiochemical parameters such as morphology, crystallinity, porosity, and ionic substitutions [57,94]. Carbonate substitutions in the structure of HA inhibit crystal growth and increase the solubility and resorption rate. Furthermore, carbonate replaces phosphate ions, which decreases the thermal stability of the apatite [57].

Carbonated and hydrated phases in bone apatite significantly differ in structure from HA. This explains the smaller crystallite size, higher solubility, and plate-like morphology in bone apatite compared to HA [24].

Both in vitro and in vivo trials have demonstrated better osteoclast resorption and bone replacement when CO_3_Ap was used instead of HA [45,95,96].

Stoichiometric HA does not dissolve passively. It requires osteoclast resorption in acidic conditions. Furthermore, its fabrication leads to a highly crystalline material that does not resemble natural bone; it can remain unchanged for more than 10 years [24].

Apatites have osteoconductive potential, the degree of which depends on the type of apatite. There is no osteoclast activity when HA materials are used, [23] and they cannot be replaced with new bone. On the other hand, bone tissue and CO_3_Ap are resorbed by osteoclasts, which create a weakly acidic environment. Under such conditions (pH 3–5), CO_3_Ap dissolves whereas HA remains stable. Biocompatibility, bioactivity, and osteoconductivity depend on the surface properties of the material, as the ions in the crystal lattice affect the surface charge and chemical reactivity [24].

The presence of carbonate ions maintains bone remodeling through dissolution–crystallization reactions [50].

There are two distinct types of biological apatite: bone apatite (with 5–8 wt% carbonate substitution) and the apatite in dental hard tissues (with 2–4 wt% carbonates in the enamel) [24,96,97]. Biomaterials used to replace damaged or missing tissues should be selected according to the characteristics and requirements of these structures [98]. Human bones consist of 55–60 wt% apatite, about 30 wt% collagen type I, and 10–15 wt% water. It is a nanocomposite in which bundles of collagen create a scaffold for the nucleation of CO_3_Ap crystallites [24]. Biological apatites experience fewer ionic substitutions than geological apatites, due to the limited amount of elements in bodily fluids [63].

The major substitution in bone apatite is by carbonate ions (5–8 wt%). There are two general types of substitution—the hydroxyl position (type A) and the phosphate position (type B)—which lead to various geometric configurations. B-type substitution influences the physical properties of the apatite, such as changes in the α- and *c*-axial lengths, crystallite size, crystallographic microstrain, optical birefringence, and mechanical strength. The increase in solubility is due to the weaker Ca–CO_3_ bonds in B-type substitution [63].

### 3.2. Carbonate Apatite

Carbonate apatite is a CPC with a similar carbonate content to that of bone apatite. It was recently utilized as a synthetic bone substitute material in bone reconstructive surgery [20].

The first in-human clinical trials were conducted in patients who underwent sinus floor elevation in three university hospitals in Japan [61,62]. The material was approved for clinical use in the country and became commercially available globally [14,59].

Although CO_3_Ap resembles natural bone, it cannot be used in powder form as a bone substitute, as it induces inflammatory reactions. Carbonate apatite has demonstrated better thermodynamical stability and reduced solubility than HA; however, it dissolves faster than HA under physiological conditions [20].

High-temperature manufacturing can enhance the strength and crystallinity of the material but reduces its bioactivity and resorption and, thus, its initial biomimetic properties. In contrast, CO_3_Ap fabricated through dissolution–precipitation reactions presents excellent tissue behavior. The processing also determines the porosity, crystallinity, surface activity, and solubility [24].

Carbonate apatite is resorbed by osteoclasts under weakly acidic conditions and replaced with new bone through bone remodeling. Its resorption rate accompanies the deposition of new bone tissue [12]. It also up-regulates osteoblast differentiation and demonstrates better osteoconductive properties than HA [59]. Carbonate apatite promotes bone deposition without fibrotic tissue formation. Furthermore, microstructural analysis has demonstrated new bone formation within the grafting material [20].

It was suggested that the osteoblast response to CO_3_Ap could serve as an indicator of osteoconductivity and that CO_3_Ap may have superior properties to other bone substitutes [20].

Modulation of osteoblast and osteoclast behavior can be achieved by altering the carbonate concentration. Therefore, the specific purpose could determine the carbonate concentration needed [24].

Carbonate substitution in HA causes lower crystallinity and improved solubility and bioactivity. Carbonate apatite is a non-toxic and biocompatible material that promotes osteoblast adhesion and proliferation [71].

The incorporation of carbonate in crystalline apatite structure changes the physiochemical properties, reduces the thermal stability, and increases the solubility of the apatite [73]. Furthermore, the increased carbonate content leads to decreases in the bulk modulus and elastic strain ratio. These findings suggest that the mechanical function of bone could be modulated and biomaterials, biocomposites, and scaffolds could be adapted to specific medical needs.

According to existing knowledge, bone apatite possesses an elastic modulus of about 60–127 GPa. These values were measured on synthetic carbonated hydroxyapatite and depended on the carbonate content [73]. It was suggested that the mechanical properties of bone apatites depend not only on the carbonate substitution but also on additional factors that have not yet been fully documented.

A method for overcoming the bio-inert behavior of metallic implants and promoting osteointegration is coating their surface with materials that mimic natural bone, in terms of composition, crystallinity, Ca/P ratio, and lattice characteristics [71].

Carbonate apatite was shown to increase bone formation around dental implants compared to HA, not only on the bone and implant surfaces but also in the center of the defect [59]. This material could serve as a coating that improves the osteoconductivity of dental and orthopedic titanium implants. It also increases bone–implant contact and adhesion strength and presents an excellent tissue response [20].

The properties of this biomaterial could be improved through co-substitutions of CO_3_ and other ions with concentrations equal to those in the physiological environment. Such co-substitutions were recently reported, using carbonate ions along with magnesium, yttrium, sodium, strontium, or silicate ions [75]. For instance, coatings of manganese-substituted CO_3_Ap on titanium promoted metabolism activation, osteoblast differentiation, and proliferation [57]. The topography of the CO_3_Ap coating also influences its properties. Smooth surfaces promote osteoclast activity, while micro-roughness hinders active ring formation [77].

Further research is necessary to establish the application of multiple-substituted HA and evaluate its biological and mechanical properties [75].

Ishikawa K. (2019) has suggested that “learning from the bone” is a successful strategy to improve the results of bone grafting. This statement corresponds to the biomimetic approaches for bone healing and restoration [14]. Biomimetic deposition aims to fabricate artificial apatite that mimics the biological apatite to improve implant osteointegration. It was demonstrated that CO_3_Ap could be successfully utilized as a biomimetic material for bone regeneration [79].

It should be noted that osteoconductivity and bone replacement depend not only on the composition but also on the structure of the bone graft. For comparison, the remodeling of cancellous bone is ten times faster than that of cortical bone. Therefore, the interconnected porous structure of bone grafts is a significant feature for rapid bone replacement [14].

Carbonate apatite was suggested as a bone substitute material for sinus floor elevation, ridge preservation, and periodontal regeneration [81].

It can be fabricated as a 3D scaffold with improved porosity, pore size, and percentage weight. An ideal scaffold should meet the following criteria: good mechanical strength and physical properties similar to those of natural bone. It should also have high osteoinductive and osteoconductive potential. The porosity and interconnectivity of the scaffold should resemble the bone structure and allow for angiogenesis. Furthermore, it should be biocompatible and demonstrate biodegradability at a rate similar to that of bone tissue. The objective of bone tissue engineering is the development of a material that replicates the mineral phase of bone. CO_3_Ap seems to be such a material [28].

Micron and mesoporous CO_3_Ap microspheres have demonstrated excellent drug-loading efficiency [57]. Several studies have demonstrated that CO_3_Ap could be used as a carrier for aminoglycosides [83]. Mesoporous microspheres of CO_3_Ap were used for controlled delivery of gentamycin and vancomycin demonstrating excellent biocompatibility and antibacterial properties [83,85], and the material successfully prevented the adhesion of *Staphylococcus epidermidis*.

In addition, CO_3_Ap coatings on implant surfaces not only improve their mechanical and biological properties but may also be loaded with bioactive molecules to serve as carriers [87].

Furthermore, CO_3_Ap could be used for the fabrication of various composite materials and hybrid scaffolds [50], allowing for the improvement of their properties and tissue behavior.

Scaffolds composed of carbonated hydroxyapatite/polysaccharide were shown to possess excellent biocompatibility, osteogenesis, and manipulation properties. They may be used as carriers for different biological molecules and medical substances [50], and such scaffolds could allow for the simultaneous release of more than one pharmaceutical substance (dual drug delivery).

Moreover, a revolutionary approach to bone regeneration is the incorporation of pro-angiogenic factors that can induce angiogenesis in the scaffold [50].

From biological and mineralogical points of view, bone apatite demonstrates both structural stability and biodegradability. Carbonate apatites could be utilized for various biomedical applications, such as bone substitution, imaging markers, scaffolding, drug delivery, and tissue engineering of biomimetic materials with improved regenerative properties [73].

## 4. Discussion

Over two million bone grafting procedures are performed annually worldwide, with bone being the second most transplanted tissue [99].

Autogenous bone grafts (autografts) are the gold standard in bone reconstructive surgery, due to their biocompatible, osteoinductive, and osteoconductive properties [13].

This means that they promote bone formation with minimal immunological response. However, they cannot be used for large bone reconstructions, due to the limited amount of donor tissues and concerns regarding postoperative morbidity [14].

Therefore, alternative bone substitutes must be applied. Synthetic bone substitutes have osteoconductive properties and feature the following advantages: abundant resources; cost-effectiveness; and no need for a donor site.

Such already available alternative materials include synthetic grafting materials, CPCs, bioactive glasses, and some biodegradable polymers. However, all of these materials present significant drawbacks and limitations [17], necessitating either their improvement or the development of new materials.

Bone grafting materials should meet the following criteria: biodegradability, biocompatibility, bioresorbability, and osteoconductivity.

**Figure 2:**
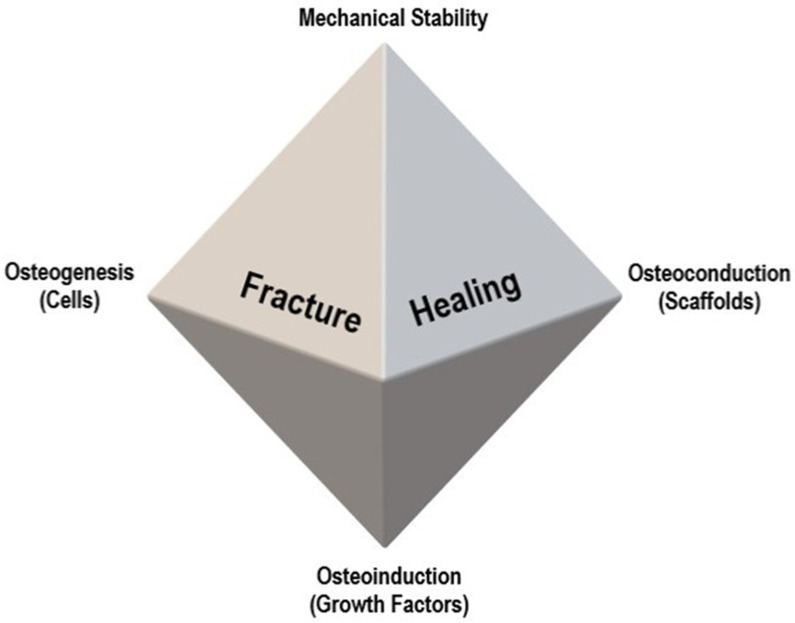
Diamond concept of fracture healing, proposed by Giannoudis et al. [100].

For a proper understanding of bone regeneration and the role of bone grafts, the diamond concept, proposed by Giannoudis et al., [100] should be considered. It demonstrates the four elements necessary for bone fracture healing: osteogenic cells, growth factors (osteoinduction), scaffolds (osteoconduction), and mechanical stability (Figure 2).

Some of the most commonly used biomaterials for bone reconstruction are HA and β-TCP. They act as scaffolds and induce bone deposition in their pores [101]. However, sintered HA exhibits numerous drawbacks, such as its long stability and acting as a foreign body. Its granules were observed to be covered with fibrous tissue two weeks after surgery and its slow resorption poses a risk for secondary infection. In addition, its elasticity differs from that of natural bone. Hydroxyapatite presents poor osteoconductive properties when compared to autologous bone [102]. As for β-TCP, it exhibits fast resorption rates, and still, the results of studies on whether the material could be fully resorbed [103,104] or whether there is a risk of inflammatory responses (similar to HA) remain heterogeneous [20].

Bone mineral belongs to the apatite series; in particular, it is carbonated non-stoichiometric, poor-crystalline, hydroxyl-deficient, and calcium-deficient (with varying Ca/P ratio) apatite, with a carbonate content in the range of 2–9 wt% [73].

A plethora of studies have focused on understanding the composition and formation of bone minerals in order to develop new bone substitutes with improved biological and mechanical properties. Biomaterials such as CO_3_Ap [41,46,47] resemble the mineral constituency of bone more accurately than stoichiometric HA and β-TCP. Some authors have stated that bone apatite is CO_3_Ap with 6–9 mass% in its structure [59].

### 4.1. Biological Properties of CO_3_Ap

Some of the articles included in this review demonstrated that CO_3_Ap has superior biological properties to HA. It was shown to present increased bone formation, better osteoconductivity, bioactivity, and bioresorbability [20,59,71,73]. Similarly, other authors have suggested that it promotes osteogenesis with minimal immunological response [46,47,74]. Clinical trials have confirmed its safety and replacement with new bone, while sintered HA remained unchanged [14,59].

Kanazawa et al. [70] compared the in vivo behavior of CO_3_Ap and sintered HA as bone substitutes for femoral and tibial osseous defects in rabbits. Both materials showed great osteoconductive properties and tissue response. No bone replacement was registered in the HA group 24 weeks after the implantation, while in the CO_3_Ap group, the material was gradually replaced with new bone; furthermore, the CO_3_Ap block had been completely resorbed within 1–1.5 years. The replacement of the CO_3_Ap block with new bone was twice as fast at the metaphyseal part of the proximal tibia than at the epiphyseal side of the distal femur, which could contribute to better blood supply in the area. The fact that the HA remained in the defect for such a long time poses a risk of infection [70,105,106,107].

Ishikawa et al. [41] conducted a study in dogs in order to compare the physical features and tissue behavior of HA, CO_3_Ap, and β-TCP as bone substitutes. The dissolution of CO_3_Ap in acidic media (pH = 5.3, similar to that in the Howship’s lacunae) was the fastest, while in a natural solution, β-TCP dissolved first. These results suggest that CO_3_Ap is stable in a physiological environment and resorbed in the Howship’s lacunae. Among the above-mentioned substitutes, CO_3_Ap showed the fastest new bone formation.

Hayashi et al. [74] fabricated three types of honeycomb blocks composed of HA, β-TCP, and CO_3_Ap, and evaluated their effects on bone formation and maturation. The macroporous composition of the blocks was similar (regular, unidirectional pores with a similar size and equal volume of macropores) and were designed to induce cell penetration and tissue ingrowth. They conducted in vitro trials with pre-osteoblast cell cultures and in vivo trials in rabbit femurs. The in vitro experiments demonstrated that CO_3_Ap was associated with almost twice greater osteoblast maturation than HA and β-TCP. The in vivo trials revealed bone maturation and material resorption at post-operative weeks 4 and 12. The CO_3_Ap blocks demonstrated markedly faster maturation than HA and β-TCP blocks, which could be due to their different resorption rates (Table 2).

These findings confirm the possible superiority of CO_3_Ap over other ceramics, which should inspire researchers in the field to conduct further investigations. It was suggested that CO_3_Ap resembles the mineral composition of bone tissue and initiates bone remodeling similar to that of natural bone. Furthermore, CO_3_Ap was shown to have a positive effect on the differentiation of osteoblasts and the expression of some early and late osteogenesis markers, such as collagen type 1, osteopontin, osteocalcin, and alkaline phosphatase [67]. Moreover, bone marrow cells cultured on CO_3_Ap demonstrated earlier osteoblastic differentiation than those cultured on HA. The response of osteoblasts to CO_3_Ap could serve as an assessment of osteoconductivity [59].

Zhang et al. [108] compared bone replacements in the dental sockets of rats after the utilization of a CO_3_Ap bone substitute and autogenous bone. The authors investigated the osteoclast precursor cell lines and evaluated bone formation using micro-computed tomography and immunohistochemical analysis. They demonstrated that bone replacement by osteoclasts after CO_3_Ap insertion resembled the process in the sockets where the autogenous bone was used. The authors even suggested that CO_3_Ap could eventually replace autologous bone as a bone substitute material.

Carbonate apatite resembles natural apatites, which are non-stoichiometric, carbonated, and calcium-deficient. Ionic substitution causes higher solubility than HA and maintains constant tissue regeneration through dissolution–crystallization reactions. Carbonate apatite provides a better osteogenic response than stoichiometric HA; however, its higher solubility reduces coating stability, which necessitates the development of additional strategies to overcome this drawback [77].

### 4.2. Medical Applications of CO_3_Ap

Carbon apatite granules yielded excellent results during simulated tests and clinical trials in Japan. Therefore, the material was approved for clinical applications in the dental field by the Pharmaceuticals and Medical Devices Agency in 2017 [14].

In 2019, Nakagawa et al. [62] conducted a clinical trial and histomorphometric assessment regarding the application of low-crystalline CO_3_Ap in two-stage sinus floor elevation and implant placement. They reported the excellent osteoconductivity and biocompatibility of the material, without any allergic or immunological response. All implants were osseointegrated and immobile at 31 months after the surgery. Their study aimed to establish the safety and efficacy of CO_3_Ap granules in sinus floor augmentation with a residual bone height between 1 and 5 mm. They performed the first histological assessment of CO_3_Ap behavior in humans and observed new bone formation around the substitute granules in all cases. The material was in direct contact with the bone tissue. The authors concluded that CO_3_Ap granules are a safe and promising material for two-stage sinus floor augmentation.

In 2021, Nagata et al. [109] published a research article comparing the use of low-crystalline CO_3_Ap with demineralized bovine bone (Bio-Oss) for sinus floor augmentation. They performed a three-dimensional analysis to measure the volume and amount of bone resorption in both groups. In particular, cone-beam computed tomography (CBCT) was performed before, immediately after, and 6 months after the surgery. Their findings demonstrated less bone resorption in the CO_3_Ap group than in the Bio-Oss group.

Carbonate apatite may be used for the fabrication of various composite materials with improved properties. Such biocomposites (e.g., CO_3_Ap–polyglycolide, CO_3_Ap–collagen, CO_3_Ap–chitosan) have recently demonstrated promising results in bone grafting [110,111,112,113].

Furthermore, some co-substitutions were recently utilized to improve the obtained material properties (e.g., carbonate, fluoride, sodium, magnesium, yttrium, or silicate ions). This co-substitution has led to enhanced bone remodeling, when compared to HA and CO_3_Ap [63,64,114,115,116,117].

Some anionic–cationic substituted apatites were investigated as well. The most common co-substitution is with Na^+^, Mg^2+^, and CO_3_^2−^ ions. The positive influence of Mg–CO_3_Ap on cell adhesion, proliferation, and metabolic activation was reported [63,118].

Moreover, CO_3_Ap possesses a massive loading potential, which provides suitable transport properties as a carrier of biologically active substances.

Keiichi et al. [69] assessed the formation of new bone after the implantation of fibroblast growth factor (FGF)-loaded porous CO_3_Ap in bone defects in rats. The micro-computed tomography showed that FGF successfully promoted bone formation.

Nagai et al. [67] demonstrated that a combination of CO_3_Ap with a sufficient amount (50 µg) of bone morphogenetic protein-2 induced osteoblastic differentiation and new bone formation. In contrast, in the groups with a small amount (5 µg) or without bone morphogenetic protein-2, no new bone formation was observed.

Coating CO_3_Ap onto various implant materials improves their mechanical strength, bioactive potential, osteoconductivity, and—when incorporated with additional bioactive substances—osteoinductivity. In addition, the mineral coating could serve as a carrier for different pharmaceutical agents, thus enhancing bone regeneration. This biomimetic approach has obtained promising results in tissue engineering [87].

**Figure 3:**
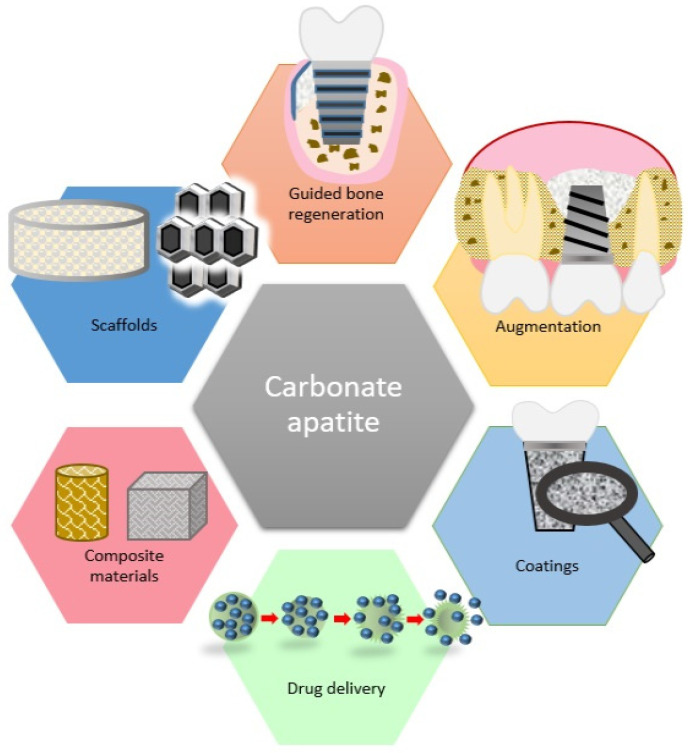
Medical applications of carbonate apatite.

It was suggested that CO_3_Ap can be utilized for various medical purposes (Figure 3). Further clinical trials are necessary to confirm their safety and efficacy.

### 4.3. Future Developments

The material science and tissue engineering fields are rapidly developing, allowing for improved bone regeneration. The developed methods involve the application of smart materials, osteogenic cells, scaffolds, and a variety of growth factors.

Carbonate apatite is a ceramic material that was demonstrated to possess promising biological properties and, thus, can be regarded as a prime candidate for a bone grafting material of choice in the future. It has demonstrated better biocompatibility, bioactivity, resorption rate, bone formation, and maturation than HA [14,20,41,59,71,73,74,105,106,107]. However, CO_3_Ap has some limitations, including its poor mechanical properties (brittleness and unsatisfactory fatigue resistance) and rapid solubility [119]. Modifications of its physicochemical characteristics (e.g., pore size, crystallinity, interconnectivity of the pores, carbonate content) [24,71,73] could successfully adapt the material for the specific needs of certain applications. This requires profound knowledge and understanding of these correlations, as well as technological preparation.

Another limitation of CO_3_Ap is its relatively expensive and technically challenging fabrication. Different technologies have been suggested, none of which have been widely adopted yet. Efforts should be directed toward the establishment of more efficient and cost-effective fabrication protocols.

Similar to the rest of the CPCs, CO_3_Ap can be used in various composite materials, including CO_3_Ap/polymers, CO_3_Ap/chitosan, CO_3_Ap/collagen, CO_3_Ap/autologous platelet concentrates, and so on. [110,111,112,113,120] As CO_3_Ap has demonstrated superior biological properties to HA and β-TCP, it is expected that this tendency will extend to the composite materials that they are also part of. Further research comparing such composite materials is needed in order to confirm or reject this hypothesis.

In addition, CO_3_Ap has demonstrated superior performance over HA and β-TCP when honeycomb blocks of these materials were tested in vivo and in vitro [74], suggesting that this material could be utilized in the processing of different standard and customized scaffolds.

Furthermore, this material has great loading potential [57]. Therefore, it should be evaluated as a carrier of biologically active substances, such as drugs, bone morphogenetic proteins, growth factors, stem cells, and so on.

Recently investigated co-substitutions of CO_3_Ap have exhibited excellent tissue behavior, cell modulation, and metabolic activation. Further experiments and clinical trials are necessary to evaluate their mechanical and biological properties.

Most of the studies in the existing literature have only reached the stage of animal trials [31,36,70,110], while little research has been conducted on the effect of CO_3_Ap on human tissues and its mechanism of action [44,61,62,109]. In this line, standardized preclinical and long-term clinical trials are necessary to establish its safety and efficacy.

These results should be evaluated using histological, histomorphometric, and CBCT analyses, which are proven diagnostic tools for the assessment of bone structures [121,122,123]. Composite materials or different coatings should be developed and researched in order to prevent rapid dissolution of the material and unwanted cell ingrowth.

It has yet to be confirmed whether CO_3_Ap may serve as a reliable biomimetic material or as a carrier in bone reconstructive surgery.

## 5. Conclusions

The increasing need for bone grafting materials with improved biological properties has led to the introduction of carbonate-containing apatites. Carbonate apatite is a calcium phosphate ceramic that resembles bone tissue with respect to reactivity, especially in acidic environments. This systematic review described and evaluated the biological properties and medical applications of CO_3_Ap.

The material was shown to possess excellent biocompatibility, bioresorbability, bioactivity, and osteoconductivity, allowing for rapid bone replacement and maturation. However, CO_3_Ap has some major limitations, such as its poor mechanical properties and high solubility. These drawbacks could be compensated for through the modification of its physicochemical properties. Therefore, the development of exact and established fabrication protocols is required.

The considered material has numerous biomedical applications and has demonstrated promising properties; as such, it may become the alloplastic material of choice for bone reconstructive surgery.

Carbonate apatite has already demonstrated its superiority over HA and β-TCP as a bone substitute and scaffold material. Furthermore, it can be used for the synthesis of biomimetic materials, composite materials, and co-substituted apatites that exhibit improved mechanical properties, biological properties, and tissue behavior.

Carbonate apatite may be utilized in tissue engineering, potentially serving as a bioactive coating and/or a drug-delivery system.

Further preclinical and long-term clinical trials are necessary to establish the safety and efficacy of this material, and to confirm its recently reported superiority over other commonly used bioceramics. The existing scientific literature has reported promising results in this regard.

## Figures and Tables

**Table 1:** Review articles included in the present study.

Reference	Year, Country	Aim	Biological Properties	Medical Applications	Conclusions Based on *
Wicramasinghe et al. [12]	2022, Sri Lanka	To summarize the current knowledge within the last 5 years and develop a novel classification of bone grafting materials.	Osteoconductivity Biocompatibility Biodegradability	Bone grafting Scaffolding	In vitro (rat cell cultures) and in vivo trials (dog model) [39] Human clinical trials [44]
Pasteris [24]	2016, USA	To discuss biological apatites from a mineralogical point of view.	Biocompatibility Bioactivity Bioresorbability Osteoconductivity	Bone grafting Scaffolding Coatings Tissue engineering	In vitro [45,46,47] and in vivo studies (rat model) [47,48]
Wu et al. [28]	2020, USA	To discuss the methods of mineralizing tissue engineering constructs.	Biocompatibility Biodegradability Osteoinductivity Osteoconductivity	Scaffolding Tissue engineering Drug delivery	In vitro trials [49]
García et al. [50]	2021, Spain	To describe different types of materials utilized in 3D scaffolds for hard tissue engineering; summarize the fabrication techniques employed to design an adequate microstructure, a hierarchical porosity (from nano- to macro-scale), a cell-friendly surface; and review the inclusion of different types of biomolecules, drugs, or cells within these scaffolds and the influence on their successful performance.	Biocompatibility Biodegradability Osteoconductivity	Scaffolding Tissue engineering Drug delivery	In vivo (rat model) [51], ex ovo (chicken embryo’s chorioallantoic membrane model) [52], and in vitro trials [53,54,55,56]
Singh et al. [57]	2020, India	To highlight the customization of desirable properties through controlling particle size, morphology, synthesis parameters, and substitution of mono/multi ions into the HAP structure to obtain a product appropriate for bone-tissue engineering and drug delivery applications.	Biocompatibility Biodegradability Osteoconductivity	Tissue engineering Drug delivery Coatings	In vitro (human osteoblasts) [58]
Ishikawa and Hayashi [59]	2021, Japan	To review the methods for fabricating carbonate apatite artificial bone and their clinical and animal results.	Biocompatibility Biodegradability Osteoconductivity	Bone substitution Bone augmentation Tissue engineering Coatings	In vivo (dog model [32] and rabbit model [60]) human clinical trials [61,62]
Šupová [63]	2015, Czech Republic	To summarize the recent knowledge on preparing substituted hydroxyapatites. The physicochemical properties of bioapatites and their substitutions with different ions are discussed.	Biocompatibility Bioactivity Bioresorbability Osteoconductivity	Bone regeneration Bioactive coatings Drug or gene delivery Biomagnetic particles and fluorescent markers	In vitro (human [64] and rabbit [65] bone marrow cells) and in vivo trials (dog model) [66]
Rahyussalim et al. [20]	2019, Indonesia	To reveal the potential of carbonate apatite as a bone substitute material.	Biocompatibility Biodegradability Osteoinductivity Osteoconductivity	Bone regeneration Drug carrier Coatings Scaffolds	In vitro (human [67,68] and rat cells [68]) and in vivo animal trials (rat [36,69] and rabbit models [31,70])
Ishikawa [14]	2019, Japan	Not clearly stated.	Biocompatibility Bioactivity Bioresorbability Osteoconductivity	Bone regeneration/ replacement	Human clinical trials [61,62]
Graziani et al. [71]	2018, Italy	To summarize the latest advances in the field of ion-substituted hydroxyapatite thin films.	Biocompatibility Bioactivity Bioresorbability Osteoconductivity	Coatings	In vitro trials (human mesenchymal stem cells) [72]
Wang et al. [73]	2023, China	To provide a profound understanding of the mineralogical account of the bone apatite mineral.	Biocompatibility Bioactivity Bioresorbability Osteoconductivity	Bone regeneration Scaffolding Tissue engineering	In vitro [46,47,74] and in vivo trials (rat, [48] rabbit, [74] and dog models [41])
Ratnayake et al. [75]	2017, New Zealand	To highlight the effects of different ionic substitutions on the chemical, physical, and biological properties of hydroxyapatite.	Biocompatibility Bioresorbability Osteoconductivity	Bone regeneration Bone augmentation Coatings	In vivo (sheep) [76] and in vitro (human osteoblasts) [64]
Arcos and Vallet-Regi [77]	2020, Spain	To review the biological behavior of substituted hydroxyapatite coatings.	Biocompatibility Osteoconductivity Biodegradability	Coatings	In vitro study (rat model [78])
Šupová [79]	2020, Czech Republic	To review methods for the synthesis of protein–calcium phosphate hybrid materials.	Not specified	Bone regeneration Scaffolding Tissue engineering Biocomposites Carriers and delivery systems	In vitro (human cells) [80]
Pajor et al. [81]	2019, Poland	To present the roles of hydroxyapatite and fluorapatite in dentistry.	Not specified	Bone regeneration Bone augmentation	Book chapter [82]
Munir et al. [83]	2021, Saudi Arabia	To summarize the applications of nano-hydroxyapatite as a delivery system of active pharmaceutical agents.	Biocompatibility Antibacterial properties	Delivery systems	In vitro study [84]
Mondal et al. [85]	2018, Russia	To review the characteristics of hydroxyapatite and nano-hydroxyapatite drug carriers.	Not specified	Drug delivery	In vitro trial [86]
Shin et al. [87]	2017, USA	To summarize the methods used to coat carbonated apatite onto various material surfaces.	Bioactivity Osteoinductivity Osteoconductivity	Bone regeneration Coatings Carriers and delivery systems	In vitro trial [88]

* Note: the references in column 6 were extracted from the corresponding review articles in column 1.

**Table 2:** Bone maturation and resorption of 3 different honeycomb blocks composed of Carbonate Apatite (CO_3_Ap), Hydroxyapatite (HA), and β-tricalcium Phosphate (β-TCP)—research by Hayashi et al. [74].

Material	Mature Bone Area (%)		Residual Material Area (%)	
	4 Weeks	12 Weeks	4 Weeks	12 Weeks
CO_3_Ap	14.3 ± 3.8	19.5 ± 0.8	73.2 ± 3.1	45.3 ± 15.9
HA	1.0 ± 0.8	2.6 ± 1.4	90.0 ± 8.9	89.5 ± 11.8
β-TCP	3.3 ± 1.2	13.7 ± 2.1	65.4 ± 2.8	7.5 ± 1.6

## Data Availability

Data were extracted to Microsoft Excel spreadsheets and processed there. The data are available on request from the corresponding author.
